# Predictors of sustained virologic response in hepatitis C genotype 4: beyond the usual suspects

**DOI:** 10.4103/0256-4947.51810

**Published:** 2009

**Authors:** Ayman A. Abdo, Faisal M. Sanai

**Affiliations:** a From the Gastroenterology Unit, Department of Medicine, College of Medicine, King Saud University, Riyadh, Saudi Arabia; b From the Hepatology Unit, Department of Medicine, Riyadh Military Hospital, Riyadh, Saudi Arabia

Treatment of chronic hepatitis C virus (HCV) has advanced considerably since the initial identification of the virus in the late 1980s. Preliminary treatment with conventional interferon monotherapy yielded disappointing sustained virologic response (SVR) rates of 10% to 15%.^1^ Since then, significant advances have been made in the treatment of HCV with reported SVR rates rising to greater than 50% with the use of pegylated interferon and ribavirin combination therapies. However, due to a variety of factors, genotype 4 patients have not been well represented in the large registry trials of antiviral therapy.^2^ This gap in medicinal wisdom has been largely filled by investigators from Saudi Arabia, Egypt and Kuwait, where genotype 4 is the predominant form, aiding us in addressing important issues relating to the management of HCV.^3^–^7^ Overall results of these trials indicate that an anticipated SVR in genotype 4 patients is around 50% to 70%.^8^

Only a minority of HCV-infected patients will develop serious disease-related complications,^9^ and hence it is this group that principally must be treated. In recommending patients for therapy, knowledge of the natural history and prognosis is important since currently available treatment regimens have limited efficacy and are difficult to tolerate. Worsening of hepatic fibrosis is the best surrogate marker of disease progression. In the absence of serial liver biopsies, the extent of serum alanine aminotransferase (ALT) elevation remains the best predictor of disease progression.^10^ However, studies from this region, where genotype 4 remains predominant, have shown that ALT levels do not predict hepatic histological findings.^11^

Predictors of response to therapy serve as decision tools for physicians to help identify patients who are likely or unlikely to achieve an SVR, and to consider pre-treatment counseling in those patients with a reduced likelihood of successful therapy, perhaps sparing them the side effects and cost of therapy. Therefore, knowledge of predictors to these therapies is extremely valuable. Traditional predictors of response identified in international studies regardless of genotype can be divided into three groups: (1) epidemiological factors including patient age, sex, and race, (2) viral factors, most importantly the pre-treatment viral load, rapid virologic response, and the genotype, and (3) histological factors including the amount of fibrosis and steatosis.^8^ In previous studies on genotype 4, some of the above predictive factors were confirmed, including age, pretreatment viral load, and stage of fibrosis.^4^,^5^,^7^

In this issue of the *Annals*, Al-Ashgar and colleagues report the results of their retrospective analysis of 148 HCV genotype 4 patients who underwent therapy with pegylated interferon alfa-2a plus ribavirin for 48 weeks.^12^ Performing a subgroup analysis of their previously published data in treating 335 patients with various HCV genotypes,^13^ the investigators report an SVR of 44.6% in the entire cohort and 50.8% in those who completed therapy in the present study of genotype 4 patients. The authors report that the predictors of response to therapy were younger age, absence of diabetes, a higher serum albumin, lower serum alpha-fetoprotein (AFP) and aspartate aminotransferase (AST) levels, and being treatment naive. On multivariate analysis, independent predictors of response were younger age, lower AST, and being treatment naive.

The study by Al-Ashgar and colleagues is significant in that it adds to the growing pool of data relating to the treatment of HCV genotype 4. It is important to note that this is the first large study from the region reporting on the efficacy of pegylated interferon alfa-2a in genotype 4 patients, in addition to the fact that it contributes essential knowledge on the predictors of virologic response. However, the study suffers from several shortcomings in design, and is hindered by unexpected and unconventional findings. The SVR observed in this study (44.6% by intention-to-treat [ITT] analysis) is certainly lower than that reported previously in genotype 4 patients using pegylated interferon alfa-2b and weight-based, standard-dose ribavirin (e.g., 69% by Kamal,^3^ 68% by Hasan,^5^ and 55% by Al Zayadi).^14^ The likely explanation for this lower SVR rate is the fact that the study group included a heterogeneous cohort of patients including post-organ transplantation, HCV-HIV co-infection, and non-responders to previous therapies, all factors known to be associated with a lower rate of response. This is unfortunate since the data concerning pegylated interferon alfa-2a in genotype 4 patients is sparse and thereby the quest for this information remains partly unfulfilled. Two previous studies^15^,^16^ (including 100 and 38 patients, respectively) using the alfa-2a form of pegylated interferon in genotype 4 patients suggest that the response rates may be higher. Obviously, any attempt at comparing treatment success between the two forms of pegylated interferons amongst this particular genotype, based on the available data, would be unfair and premature at best, due to the lack of large, prospectively conducted studies using the alfa-2a form of pegylated interferon.

The second important finding of this study is related to the predictors of response, where traditional predictors like pre-treatment viral load and extent of hepatic fibrosis fell by the wayside, and non-conventional factors like AST and AFP were identified to be predictive. In an earlier study of 250 Egyptian genotype 4 patients, the presence of severe fibrosis, hepatic steatosis, treatment with conventional interferon, and AFP level were found to predict SVR.^17^ The findings of the present study further augment the pool of evidence citing a higher AFP level with a negative treatment outcome.^15^,^17^,^18^ In genotype 4 patients, treatment with both alfa-2a and alfa-2b forms of pegylated interferon has shown lower AFP levels to be predictive of SVR.^17^ Similar findings have been found in genotype 1 patients.^18^ Higher serum AFP levels, in essence, may serve as surrogate markers of more advanced fibrosis^19^,^20^ and hence, the finding of the present study that advanced fibrosis is not a predictor of SVR is counterintuitive. Nonetheless, the growing recognition of AFP as a predictor of response, given its uniqueness, mandates further evaluation.

An important lapse of this retrospective analysis is that the authors did not have adequate information on the form of antiviral therapy administered previously, whether it was conventional interferon vs. pegylated form, monotherapy vs. combination with ribavirin, or the duration of such therapy. Also, as has been convincingly demonstrated in earlier trials, a nonresponder to, or relapser after, previous antiviral therapy significantly affects the likelihood of response to subsequent interferon-based therapy. All such pertinent information is unavailable in the current study and may have affected the results of the univariate analysis. Moreover, because of the retrospective nature of the study, only 72 of the 96 patients who were analyzed had a pre-treatment liver biopsy. This, in part, may explain why none of the histological predictors were identified as opposed to almost all other genotype 4 and non-genotype 4 studies. Thus, the unavailability of this information in the current study may likely have affected the results of the univariate analysis, and in effect limits the overall scope of the study.

Finally, the authors, while reporting SVR rates with an ITT methodology, did not maintain this while performing the regression analysis. ITT analysis denotes analyzing patients in the groups they were assigned to at the beginning of the study regardless of whether they actually completed therapy or not. Deviations from ITT analysis introduce potential investigator bias in interpretation of results with the inherent plausibility of overestimating drug efficacy.

Despite these concerns, this study remains an important contribution to our understanding of virologic response in genotype 4 patients. Future studies should be directed at investigating the optimal duration of therapy utilizing the rapid virologic response and early viral kinetics.^8^ In addition, the utility of AFP as a predictor of response and possibly as an indicator of liver fibrosis and/or inflammation is worth further evaluation. Moreover, the role of the newer “small antiviral molecules” in genotype 4 patients, either in isolation or in combination with pegylated interferon, needs to be imminently studied. Physicians may then use the available data on predictors of response to interferon-based therapy to better direct the choice between the various treatment options in order to tailor therapy to individual patients both, in relation to the type of therapy used and its duration.

Major advances in the treatment of HCV in the last decade offer us hope that the day of curing all patients with HCV is not too far away. While we stand one step closer to our destiny today, our goal per se is aptly exemplified in the words of T.S Eliot: “Only those who risk going too far can possibly find out how far one can go.” And this time HCV patients with genotype 4 should journey the whole length and not be left behind.

## References

[1] Lauer G, Walker B (2001). Hepatitis C virus infection. N Engl J Med.

[2] Abdo AA, Lee SS (2004). Management of hepatitis C virus genotype 4. J Gastroenterol Hepatol.

[3] Al-Faleh FZ, Aljumah A, Rezeig M, Al-Kanawi, Al-Otaibi M, Alahdal M, Al-Humayed S, Mayet I, Al-Juhani M, Al-Karawi M, George K, Sbeih F (2000). Treatment of chronic hepatitis C genotype 4 with interferon ribavirin combination in Saudi Arabia: a multicenter study. J Viral Hep.

[4] Alfaleh FZ, Hadad Q, Khuroo MS, Aljumah A, Algamedi A, Alashgar H, Al-Ahdal MN, Mayet I, Khan MQ, Kessie G (2004). Peginterferon alpha-2b plus ribavirin compared with peginterefron alpha 2b for initial treatment of chronic hepatitis C in Saudi patients commonly infected with genotype 4. Liver Int.

[5] Kamal SM, El Tawil AA, Nakano T, He Q, Rasenack J, Hakam SA, Saleh WA, Ismail A, Aziz AA, Madwar MA (2005). Peginterferon alpha 2b and ribavirin therapy in chronic hepatitis C genotype 4: impact of treatment duration and viral kinetics on sustained virological response. Gut.

[6] Kamal S, Al Kamary S, Shardell, Hashem M, Ahmed I, Muhammadi M, Sayed K (2007). Pegylated interferon alpha 2b plus ribavirin in patients with genotype 4 chronic hepatitis C: the role of rapid and early virological response. Hepatology.

[7] Hasan F, Asker H, AL Khaldi J, Siddique I, AL Ajmi M, Owaid S, Varghese R, Al-Nakib B (2004). Peginterferon alfa 2b plus ribavirin for the treatment of chronic hepatitis C genotype 4. Am J Gastroenterol.

[8] Kamal S, Nasser I (2008). Hepatitis C genotype 4: what we know and we don't yet know. Hepatology.

[9] Seeff LB, Hollinger FB, Alter HJ, Wright EC, Cain CM, Buskell ZJ, Ishak KG, Iber FL, Toro D, Samanta A, Koretz RL, Perriollo RP (2001). Long-term mortality and morbidity of transfusion-associated non-A, non-B, and type C hepatitis: A National Heart, Lung, and Blood Institute collaborative study. Hepatology.

[10] Ghany MG, Kleiner DE, Alter H, Doo E, Khokar F, Promrat K, Herion D, Park Y, Liang TJ, Hoofnagle JH (2003). Progression of fibrosis in chronic hepatitis C. Gastroenterology.

[11] Sanai FM, Benmousa A, Al-Hussaini H, Ashraf S, Alhafi O, Abdo AA, Alameri HF, Akbar HO, Bzeizi KI (2008). Is serum alanine transaminase level a reliable marker of histological disease in chronic hepatitis C infection?. Liver Int.

[12] Al Ashgar H, Helmy A, Khan M, Al Kahtani K, Al Quaiz M, Rezeig M (2009). Predictors of sustained virological response to a 48-week course of pegylated interfon alfa-2^a^ and ribavirin in patients infected with hepatitis C virus genotype 4. Ann Saudi Med.

[13] Al Ashgar H, Khan M, Helmy A, Al Swat K, Al Shehri A, Al Kalbani A, Peedikayel M, Al Kahtani K, Al Quaiz M, Rezeig M, Kagevi I, Al Fadda M (2008). Sustained virological response to peginterferon alpha-2^a^ and ribavirin in 335 patients with chronic hepatitis C: A tertiary care center experience. Saudi J Gastroenterol.

[14] El-Zayadi AR, Attia M, Barakat EM, Badran HM, Hamdy H, El-Tawil A, El-Nakeeb A, Selim O, Saied A (2005). Response of hepatitits C genotype 4 naive patients to 24 weeks of peginterferon alpha-2b ribavirin or induction dose interferon alpha-2b ribavirin amantadine: a non-randomized controlled study. Am J Gastroenterol.

[15] Males S, Gad RR, Esmat G, Abobakr H, Anwar M, Rekacewicz C, El Hoseiny M, Zalata K, Abdel-Hamid M, Bedossa P, Pol S, Mohamed MK, Fontanet A (2007). Serum alpha-foetoprotein level predicts treatment outcome in chronic hepatitis C. Antivir Ther.

[16] Derbala MF, Al Kaabi SR, El Dweik NZ, Pasic F, Butt MT, Yakoob R, Al-Marri A, Amer AM, Morad N, Bener A (2006). Treatment of hepatitis C virus genotype 4 with peginterferon alfa-2^a^: impact of bilharziasis and fibrosis stage. World J Gastro.

[17] Gad RR, Males S, El Makhzangy H, Shouman S, Hasan A, Attala M, El Hoseiny M, Zalata K, Abdel-Hamid M, Fontanet A, Mohamed MK, Esmat G (2008). Predictors of sustained virological response in patients with genotype 4 chronic hepatitis C. Liver Int.

[18] Akuta N, Suzuki F, Kawamura Y, Yatsuji H, Sezaki H, Suzuki Y, Hosaka T, Kobayashi M, Kobbayashi M, Arase Y, Ikeda K, Kumada H (2007). Predictors of viral kinetics to peginterferon plus ribavirin combination therapy in Japanese patients infected with hepatitis C virus genotype 1b. J Med Virol.

[19] Murashima S, Tanaka M, Haramaki M, Yutani S, Nakashima Y, Harada K, Ide T, Kumashiro R, Sata M (2006). A decrease in AFP level related to administration of interferon in patients with chronic hepatitis C and a high level of AFP. Dig Dis Sci.

[20] Chen TM, Huang PT, Tsai MH, Lin LF, Liu CC, Ho KS, Siauw CP, Chao PL, Tung JN (2007). Predictors of alpha-fetoprotein elevation in patients with chronic hepatitis C, but not hepatocellular carcinoma, and its normalization after pegylated interferon alfa 2^a^-ribavirin combination therapy. J Gastroenterol Hepatol.

